# A Cross-Sectional Study on Public Awareness About Child Sexual Abuse in Makkah, Saudi Arabia

**DOI:** 10.7759/cureus.34931

**Published:** 2023-02-13

**Authors:** Ibrahim Alharbi, Esraa I Qashgry, Reem A Almalki, Raghad A Qari, Esraa E Yassen, Sarah M Alhadhrami

**Affiliations:** 1 Pediatrics, Hematology Oncology, King Fahad Armed Forces Hospital, Jeddah, SAU; 2 Department of Medicine, Faculty of Medicine, Umm Al Qura University, Makkah, SAU

**Keywords:** practice, attitude, kap (knowledge, awareness, community, effect of sexual abuse, sexual behavior, child sexual abuse

## Abstract

Background: Child sexual abuse is a very serious and dangerous problem. It has long-term consequences and may impact children and follow them into adulthood. Without exception, regardless of their cultures and religious beliefs, all communities are vulnerable to child sexual abuse (CSA).

Objective: Our aim in this study is to assess the level of public (caregivers) awareness of CSA in Makkah and Jeddah, Saudi Arabia.

Methodology: A cross-sectional study was conducted in Makkah and Jeddah. The target populations were Saudi parents, caregivers, and teachers who are in daily contact with children. The participants were selected randomly (n=417), and were requested to complete a questionnaire that was later analyzed using the IBM Corp. Released 2015. IBM SPSS Statistics for Windows, Version 23.0. Armonk, NY: IBM Corp.

Results: 369 from 417 responders were females (88.5%), while 48 were males (11.5%). About 90.2% of the responders acknowledged the existence of the child sexual abuse problem. About 95.7% of the caregivers were aware of physical, social, and psychological consequences of child sexual abuse. In contrast, only 33.8% of the responders were aware of existence of social agencies and organizations that provide supportive services to the victims of child sexual abuse.

Conclusion: In conclusion, the study found a high rate of awareness in the caregivers who recognize the problem of child sexual abuse (CSA) and are aware of its magnitude and impact on children's well-being. However, these results demonstrate the need for more education of the public about the mandatory laws regarding child sexual abuse.

## Introduction

Child sexual abuse is a serious problem in public health that causes concerns worldwide. It's often not talked about, and in many times, it is even a forbidden issue to mention or address. According to the World Health Organization (WHO) data, there are around 40 million children every year who are affected by sexual abuse [1]. Not many studies address this sensitive issue in Saudi Arabia, especially the western region of the country.

Child sexual abuse was also present and widespread in the past [2]. Because of limited communication tools back then, this issue was somewhat hidden from the public and did not gather much attention. Now, there is more public awareness about CSA owing to social media. Even the new generations have far better awareness and understanding about it. Additionally, they are better informed on how to respond to children who have been the victims of such horrible crimes and about all types of child abuse, including sexual abuse.

The world deals with child sexual abuse in different ways according to their cultural, religious, and societal norms. In our community, it’s usually a problem surrounded by silence because of fear of shame and the perceived scandalous nature of this problem. For this reason, some families still struggle to stop sexual abuse even after educating their children about it. There are many reasons for this, such as excessive shyness or fear of the consequences. Some families are also concerned about how well their children will understand what is allowed and what is not. Some other families are worried that if they fully educate their children about sexual abuse at a young age, these children may develop an excessive sexual awareness, which may make them deviate from the righteous path emotionally and intellectually [3].

Child sexual abuse is a serious problem that should be addressed from multiple aspects. One of these important aspects is the awareness of the child who is being sexually abused of what is happening to him/her and that this is a violation of their basic rights. Once they realize this, they start to doubt themselves and develop fear as well as anxiety and it may have an impact on their personalities and their entire well-being.

A child who is a victim of sexual abuse may be unable to handle the abuse and, at the same time, is unable to express themselves and talk about what happened to them as well. Subsequently, they are prone to develop certain symptoms such as anxiety, excessive worry, and feeling fearful [4]. They may blame themselves and become regretful, which may result in them being unable to be truthful with their parents. Reasons for being unable to talk to their parents or caregivers are fear of punishment, feeling ashamed, and worry about society's view of them. These conflicts may generate psychological problems such as isolating themselves from society, fear of going out of the house, fear of going to school, being unable to have eye contact, and other problems as well [5].

Some families deal with the problem in the wrong way, such as concealing the problem, denying it, not accepting the matter, or not taking it seriously [6]. One reason is that sexual abuse often does not have obvious physical marks, which leads to the parents and caregivers denying it or even dismissing the incident and considering it as if it did not happen.

One of the difficulties that make child sexual abuse cases less reportable is the conservative environment in which the child and his family live. Conservative societies are usually so secretive and tend to hide such incidents. Family ignorance of the child’s level of awareness that is optimum to his/her age and not taking what they say seriously. Also, the presence of uneducated parents of less aware or poorly conscientious of what their children say [4]. 

## Materials and methods

A cross-sectional study was conducted in Makkah and Jeddah regions between December 10, 2020, and March 10, 2021. The target populations were Saudi parents, caregivers, and teachers who are in daily contact with children.

Individuals who had training or knowledge about child sexual abuse or people who work in child protection organizations were excluded. Based on the population of Makkah and Jeddah regions, according to the general authority for statistics, a sample size of 385 individuals was calculated by using the Raosoft sample size calculator as enough to produce a 95% confidence interval with a 5% accepted margin of error when the estimated proportion of child sexual abuse (CSA) was 50.0%. A simple random sampling technique is used to collect the data in which each sample has an equal probability of being chosen.

The data was obtained through multiple settings (malls, hospitals) and social media (Twitter, WhatsApp) using self-administered questionnaires consisting of closed-ended questions.

The data analysis was done using the IBM Corp. Released 2015. IBM SPSS Statistics for Windows, Version 23.0. Armonk, NY: IBM Corp. Descriptive statistics, including percentages, mean, median, mode, range, variance, SD, and frequency, were taken into consideration, and distribution for each variable was calculated.

## Results

Statistical analysis

All variables were categorical and therefore presented as frequencies and percentages. Points were assigned to 23 Likert scale questions such as: disagree = 1, somewhat disagree = 2, I don’t know = 3, somewhat agree = 4, and agree = 5. The total knowledge, attitude, and practice (KAP) scores were calculated by adding these points for all 23 questions. The total KAP score was compared between males and females by the Mann-Whitney U test. The former was compared to other socio-demographic variables (age, education, job, income, and marital status) by the Kruskal-Wallis H test.

The analysis was performed in a 95% confidence interval using the IBM Corp. Released 2015. IBM SPSS Statistics for Windows, Version 23.0. Armonk, NY: IBM Corp.

Results

A total of 417 child caregivers were interviewed in this study. The majority of them were females (369.417, approximately 85.5%). The average age between 20 and 30 years is found in 221 (53.0%). Most of them were from Makkah (69.5%) since the study was mainly conducted in Makkah city. More than two-thirds of the respondents had a college degree (66.9%). About 7.2% of responders have a post-graduate degree. When it comes to the socioeconomic status of the respondents, many of them have an average income (87.3%), and almost half of them are married (50.6%) (Table 1).

**Table 1:** Sociodemographic characteristics of all respondents (n = 417)

Characteristics	N	%
Gender		
Female	369	88.5
Male	48	11.5
Age		
20 – 30	221	53.0
31 – 40	76	18.2
41 – 50	70	16.8
> 50	50	12.0
Residence		
Makkah	290	69.5
Jeddah	110	26.4
Riyadh	4	1.0
Yanbu	4	1.0
Medina	3	.7
Al Qunfudhah	2	.5
Taif	2	.5
Dhahran	1	.2
Tabuk	1	.2

Education		
Primary	3	.7
Secondary	7	1.7
High School	98	23.5
University	279	66.9
Postgraduate Studies	30	7.2
Job		
Student	156	37.4
Employed	96	23.0
Unemployed	140	33.6
Retired	25	6.0
Income		
Low	28	6.7
Medium	364	87.3
High	25	6.0
Marital status		
Single	183	43.9
Married	211	50.6
Divorced	17	4.1
Widow	6	1.4

About 90.2% of caregivers interviewed knew that child sexual abuse is a major problem around the world. When it comes to the consequences of child sexual abuse, 95.7% of the caregivers interviewed acknowledged the statement that “CSA has unwanted and even detrimental physical, social, and psychological consequences. In contrast, only 33.8 % of the responders were aware of the existence of social agencies and organizations that provide supportive services to the victims of child sexual abuse. All 23 CSA questions and answers were presented in Table 3. Table 2 shows the mean and standard deviation of this Likert scale data. The highest mean was observed for Q1 (4.87 ± 0.445), and the lowest mean was seen for Q12 (3.66 ± 1.162) (Tables 2, 3). 

**Table 2:** Mean and standard deviation of child sexual abuse related Likert Scale variables

Q. No.	Questions	Mean	SD
1	Child sexual abuse problem exists around the world.	4.87	.445
2	Children are more likely to be sexually abused by Their relatives\someone the child know and trusts.	4.33	.877
3	A person who has sexually abused as a child will likely repeat the offense.	3.85	1.180
4	Usually there are no obvious physical evidence of a child who sexually abused.	3.57	1.254
5	Females cannot be sexually abuse children.	2.63	1.434
6	Boys cannot be sexually abused.	1.33	.731
7	All children who report or disclose CSA are telling the truth.	3.74	1.238
8	Men sexually abuse children in most cases.	4.16	1.130
9	The child who was sexually abused is not to be blamed.	4.41	1.025
10	The problems of CSA have physical, social, and psychological negative consequences.	4.95	.250
11	There are mandatory laws that protect children from CSA.	3.81	1.238
12	There are social organizations that provide services for children exposed to CSA.	3.66	1.162
13	Does the child have a fear of being alone with a certain person?	4.60	.704
14	Does the child have any emotional or behavioral changes?	4.71	.593
15	Does the child feel pain while passing urine or stool?	4.26	.912
16	Did you notice any unexplained bruises in unusual sites?	4.24	.921
17	Does the child recently urinate in unusual places?	3.92	.988
18	Did the child become lonely and isolated.	4.39	.887
19	Educated caregivers know more about CSA than non- educated or undereducated mothers.	4.18	.995
20	Caregivers educate their children about CSA more than fathers.	4.14	1.068
21	The children should be taught about CSA in early age.	4.76	.652
22	Children’s education about CSA can prevent CSA.	4.40	.818
23	Caregivers knowledge about CSA can prevent the CSA.	4.25	.960

**Table 3:** Likert scale variables presented as percentages

Questions	Disagree	Somewhat disagree	I don’t know	Somewhat agree	Agree
Q1	Nil	0.5	2.6	6.7	90.2
Q2	1.0	4.6	7.9	33.8	52.8
Q3	6.0	7.7	18.9	30.0	37.4
Q4	7.9	14.1	19.7	30.0	28.3
Q5	31.2	20.6	16.8	17.3	14.1
Q6	77.5	16.1	3.4	2.2	1.0
Q7	5.3	15.1	15.6	28.1	36.0
Q8	4.3	8.2	6.5	29.0	52.0
Q9	2.4	6.7	5.8	17.5	67.6
Q10	Nil	Nil	0.7	3.6	95.7
Q11	6.7	6.5	28.3	16.5	42.0
Q12	6.2	3.6	42.2	14.1	33.8
Q13	0.5	1.0	7.0	21.1	70.5
Q14	0.2	0.2	5.0	17.5	77.0
Q15	0.5	0.5	27.6	15.6	55.9
Q16	1.0	1.9	21.6	22.8	52.8
Q17	1.25	1.4	41.7	15.8	39.8
Q18	1.0	2.9	12.9	22.8	60.4
Q19	1.7	4.3	18.9	24.2	50.8
Q20	3.1	7.7	9.1	32.1	48.0
Q21	1.2	0.7	2.6	12.2	83.2
Q22	1.2	3.4	4.1	36.7	54.7
Q23	1.7	3.1	17.3	24.7	53.2

The mean total KAP score for all caregivers was 93.1463 ± 8.02911. Females (93.6287 ± 7.76841) had statistically significantly higher mean scores than males (89.4375 ± 9.06691), p-value = .002. (Tables 4, 5).

**Table 4:** Total score

Measurements	Values
Mean	93.1463
Median	94.0000
Std. Deviation	8.02911

**Table 5:** Relationship between gender and total score

Gender	Mean ± SD of total score	p-value
Male	89.4375 ± 9.06691	.002
Female	93.6287 ± 7.76841	

The relationship between education level and total KAP score was also significant, with p-value = .003. The relationship between age and KAP of CSA score was significant, with p-value = .003. A significant relationship between marital status and KAP score was also seen, with p-value = <.001. However, the relationship between income, employment, and total KAP score was not statistically significant, with p-values of .172 and .472, respectively (Table 6).

**Table 6:** Relationship between education, age, income, marital status, employment and total score

Education	Mean ± SD of total score	p-value
Primary	78.3333 ± 9.01850	.003
Secondary	101.7143 ± 6.15668	
High-school	94.1224 ± 7.96547	
University	92.8029 ± 7.92777	
Postgraduate	92.6333 ± 7.17987	
Age		
20 - 30	92.3258 ± 7.60100	.003
31 - 40	93.3684 ± 8.87820	
41 - 50	94.3143 ± 8.80965	
> 50	94.8000 ± 7.07972	
Income		
Low	90.2143 ± 8.66392	.172
Medium	93.4258 ± 7.98880	
High	92.3600 ± 7.47150	
Marital status		
Single	91.6721 ± 7.73473	< .001
Married	94.0000 ± 8.09056	
Divorced	99.2353 ± 5.80441	
Widow	90.8333 ± 9.28260	
Employment		
Student	92.6154 ± 7.49749	.472
Employed	93.7813 ± 8.49297	
Unemployed	93.0857 ± 8.47042	
Retired	94.3600 ± 6.95150	

## Discussion

World Health Organization (WHO) has defined Child Sexual Abuse (CSA) as “the involvement of a child in sexual activity that he or she does not fully comprehend, is unable to give informed consent to, or for which the child is not developmentally prepared and cannot give consent, or that violates the laws or social taboos of society”. Child sexual abuse usually happens between a child and an adult or a child and another older child. Both the adult and the older child are usually persons of trust, power, or direct or indirect responsibility for the child being abused. The abusive activity is meant to gain sexual gratification. Child sexual abuse has existed throughout human history [7]. 

Child sexual abuse is a major problem in the world today. Many reasons contribute to this widespread problem. First is the vulnerability of children. Second, different circumstances put children at greater risk for sexual abuse, such as the absence of parental supervision, poverty and child labor, family dysfunction, stressful home environment, unmonitored access to social media websites and applications, violence in the family, and low self-esteem [8]. 

The dynamics of child sexual abuse are very important for healthcare professionals and caregivers as well. First, in most cases, the perpetrator is well-known to the child or a relative/family member. Indeed, one-third of confirmed child sexual abuse usually involves a family member. Second, violence and force are rarely used in cases of confirmed child sexual abuse. The perpetrator usually uses different tactics; coercion, manipulation, guilt inciting, and other ways to convince the victim. Third, child sexual abuse is usually repetitive and occurs over a long period that may extend into years. Fourth, child sexual abuse usually becomes more intrusive with time. Understanding these facts about the nature of child sexual abuse is very important in recognizing the cases [9]. 

The prevalence of this problem differs from one society to another. Nonetheless, most societies suffer from child sexual abuse to varying degrees [10]. It was not till the 1970s and 1980s that public awareness of child sexual abuse became more evident, and laws and regulations have been made to protect children, provide care for the victims of these acts, and took legal measures against the perpetrators [11].

The reason we conducted this study is that many childhood sexual abuse victims are suffering in silence. They are afraid to come forward and disclose what they are going through. It is important to measure parental/caregiver awareness of this problem. Child sexual abuse is underreported in today’s societies. This makes the problem even worse as many of these events are not recognized, and, therefore, these children do not receive the proper interventions that they need to help them deal with it and minimize the consequences of such crimes. As medical professionals, we play an important role in educating, supporting, and managing such children who are victims of this type of abuse and their families [12].

In our study, we tried to find out the parental and caregiver’s knowledge and attitudes toward child sexual abuse. While the majority were aware of the fact that child sexual abuse is a major internationally recognized problem in the world today and was able to recognize the consequences of such a devastating problem, only a few (33%) recognized that there are social agencies and government organizations that can help them when encountering such a problem. We also found that when it comes to KAP scores, females are better in terms of understanding the issues related to child sexual abuse compared to male caregivers. Probably this is because our society is conservative and, in general, women are closer to their children than their male counterparts. 

There were also variations in KAP scores among caregivers when it comes to the age of the caregivers. Older parents and caregivers, specifically older than 50, have a higher score than younger couples. This is probably because older couples and caregivers have much more experience with raising children than younger and new couples. This difference is statistically significant and relevant as older couples are more capable of dealing with cases of CSA than young couples. Also, young couples are usually in the process of building themselves, so many times, they are preoccupied to be present with, close to, and fully aware of their children’s problems.

Our study also revealed that, in addition to the key ones, other factors affect caregivers' overall KAP scores and, consequently, their knowledge of child sexual abuse. When it comes to marital status, parents and caregivers who are divorced have the highest and statistically significant KAP score compared to all other social status categories. On the other hand, widows and widowers have the least KAP Score. This is an interesting finding because prior studies found the prevalence of child sexual abuse confirmed cases, and allegations of child sexual abuse occur at a higher rate in high-conflict divorces (Divorces that involve court battles and custody claims as well as financial conflicts). Probably that is why in our study, divorces were more aware of childhood sexual abuse because they anticipate the occurrence of such problems since there will be a change in the child’s sleeping locations in shared custody cases [13].

Finally, and contrary to previous studies’ findings, we did not find a statistically significant correlation between a high KAP Score and the employment status and income of the caregivers. While there are prior and recent studies linking the employment status of the caregiver with an increased risk of child abuse, many of these studies concluded that physical as well as other forms of non-sexual abuse occur more frequently in a relationship with the caregiver’s employment and income status [14].

## Conclusions

In conclusion, the study found a high rate of awareness in the caregivers who recognize the problem of child sexual abuse (CSA) and are aware of its magnitude and impact on children's well-being. However, these results demonstrate the need of raising public awareness of the mandatory laws regarding child sexual abuse. Additionally,, demonstrating how significant social and organizational support is. A lack of awareness is a barrier to recognition and reporting such cases. Additionally, there are societal traditions that may prevent people from reporting these cases, which leads to underreporting. There is a need to improve the general knowledge about CSA manifestations and preventative strategies. Therefore, special attention and further intervention tools must be implemented. In this regard, we emphasize the necessity to start educational initiatives for caregivers. We recommend more extensive research and studies to assess the prevalence of child sexual abuse in Saudi Arabia.
